# Dentine Surface Morphology after Chlorhexidine Application—SEM Study

**DOI:** 10.3390/polym10080905

**Published:** 2018-08-11

**Authors:** Barbara Lapinska, Leszek Klimek, Jerzy Sokolowski, Monika Lukomska-Szymanska

**Affiliations:** 1 Department of General Dentistry, Medical University of Lodz, 92-213 Lodz, Poland; barbara.lapinska@umed.lodz.pl (B.L.); jerzy.sokolowski@umed.lodz.pl (J.S.); 2 Department of Dental Technology, Medical University of Lodz, 92-213 Lodz, Poland; leszek.klimek@umed.lodz.pl

**Keywords:** dentine, chlorhexidine, SEM, adhesion, surface

## Abstract

Chlorhexidine (CHX) is a widely known and a very popular antibacterial agent that decreases the level of cariogenic bacteria. CHX applied on the cavity surface of dentine may influence adhesive bond strength. The aim of the study was to evaluate the dentine surface after different chlorhexidine digluconate (CHG) application protocols. Different CHG application protocols were introduced. A concentration of 0.2% or 2.0% CHG was applied on the etched or unetched dentine surface for 15 or 30 s, then water rinsed or drained. Scanning electron microscopy (SEM) observations and energy disperse spectrometer (EDS) analysis of the dentine surfaces were performed. The application of 0.2% CHG for 15 s, followed by draining, on either etched or unetched dentine surface effectively removed the smear layer, leaving the surface enriched with CHG deposits. Conclusions: The concentration of CHG and its application time influenced the amount of CHG deposits and the degree of smear layer removal from the dentine surface.

## 1. Introduction

Modern restorative dentistry (i.e., composite and all-ceramic restorations) relies on adhesion. The tooth-composite interface is the integral part of the adhesive bond. The most important substrate is dentine; however, the durability still remains a challenge. Resin tags penetrate into dental tubules, simultaneously creating a hybrid layer. Nevertheless, this bond is very stable just upon polymerization, although it is vulnerable to extreme conditions in the oral cavity. As a result, the bond strength gradually decreases, hybrid layer degradation and microleakage may occur, or bacteria remaining on the dentine surface or in dental tubules may cause secondary caries. To prevent these complications, bonding procedures were modified. On the one hand, improved formulas were implemented into ingredients of bonding agents, with the aim to exert prolonged antibacterial effect or guarantee durable bond interface. On the other hand, several agents inhibiting bacterial growth or matrix metalloproteinases were introduced. These media are applied prior or during adhesive procedures on the tooth surface.

Chlorhexidine (CHX) is a widely known and a very popular antibacterial agent that decreases the level of cariogenic bacteria in both saliva and dental plaque [1]. Rinsing with 2.0% CHX for 2 min was shown to remove *E. faecalis* in the first 100 μm of dentinal tubules [2,3].

The efficacy of CHX is reported to be associated with its substantivity [4]. The substantivity of CHX to human dentine depends on the concentration used [5]. In higher concentrations, CHX diminishes bacterial growth and penetration into dentinal tubules [6]. In lower concentrations, CHX causes a decrease in bacteria metabolism by inhibiting the activity of proteolytic and glycosidic enzymes [7].

Prolonged antibacterial activity (up to 12 weeks) results from absorption and slow release of CHX from hydroxyapatites [1]. Chlorhexidine can be chemisorbed on the surface of tooth hydroxyapatite or react as an ion, creating insoluble compounds with phosphate ions of the hydroxyapatite [8].

As binding capacity of CHX to demineralized dentine is greater than to the mineralized one, it may be presumed that the absorption mechanism of CHX is different to both types of dentine [9]. Positive charges of CHX are probably electrostatically attracted to negative charges of trivalent phosphate anion in crystal matrix [10] of demineralized dentine hydroxyapatite [9]. In the case of demineralized dentine, CHX can bind electrostatically with negatively charged carboxyl groups of collagen or create hydrogen bonds with carboxyl groups. Both types of bonds are nonspecific [9]. More than eight-fold higher absorption of CHX by demineralized dentine in comparison to mineralized dentine supports the idea of CHX application on etched dentine surface [11,12,13], followed by drying the surface, without rinsing.

It was reported that acid-etched enamel and dentine should be thoroughly rinsed to remove all reaction products. Moreover, water allows for full expansion of the demineralized dentine matrix [14]. If the excess water is not removed by blotting or a half second air blast just prior to bonding, the residual water may induce phase changes in etch-and-rinse adhesives that contain BisGMA [15,16].

Treating acid-etched dentine with 0.2–2.0 wt % CHX in water or ethanol was reported to eliminate any bacteria that survive acid-etching [9,17]. Next, CHX binds to acid-etched dentine, where it inhibits dentine matrix metalloproteinases (MMPs) and prolongs the durability of resin-dentin bonds [12]. Since acid-etched, water-rinsed, intertubular dentine matrix is composed of 70% water and 30% organic matrix, application of almost any disinfecting agent will result in its diffusion into that unbound water and then binding to the organic matrix. Such a process/reaction has recently been shown for CHX [9]. CHX is a positively charged molecule that binds to negatively charged, demineralized dentine matrix.

Another advantage of such CHX application is its rehydration capacity and high affinity to dental tissues [18]. Therefore, this compound can be used for rehydration of etched dentine surface, before application of a total-etch (TE) system, allowing for re-expansion of collagen fibrils and their recovery to primary dimensions. Breschi et al. [19] used 2.0% CHX instead of water to wet demineralized dentine surface after etching (without rinsing), which positively affected stabilization of the adhesive bonding evaluated by the μTBS test [20].

It is said that CHX incorporated into an adhesive interface may be released without altering its quality [8]. Using high concentrations of CHX on etched dentine can result in oversaturation of the substrate and initial rapid release of CHX excess. These excesses may be caught between collagen fibrils after filling the intrafibrillar space with resin during adhesive procedures, just before the resin is polymerized [21]. The adhesive, together with incorporated CHX, creates, upon polymerization, the deposit that slowly releases the disinfecting agent [22,23,24]. Such management allows for preservation of collagen fibril continuity in the hybrid layer [25,26].

Chlorhexidine (digluconate and acetate) is a synthetic, non-specific inhibitor of matrix metalloproteinases [19] MMP-2, MMP-8, and MMP-9, even at very low (0.01%–0.02%) concentrations [9,27,28,29]. Moreover, chlorhexidine digluconate applied on etched dentine inhibits MMP revealed by orthophosphoric acid.

CHX applied on the cavity surface may influence adhesive bond strength. However, there is no consensus on the way it influences the bond strength; some researchers claim that it adversely affects the bond [30,31,32,33], while others do not confirm these results [34,35,36]. According to other studies, application of CHX after etching did not influence the immediate bond strength (TBS, SBS, and μTBS) in comparison to control group [11,18,32,34,37].

Unfortunately, the behavior of CHX on the dentine surface has not been extensively investigated. There are many questions to be answered; for example, whether CHX may remain on the dentine surface upon etching, influence the etching pattern, create deposits on the dentine surface, or penetrate into dentinal tubules. Additionally, the effect of antibacterial agents on bonding properties of dental bonding agents (DBA) and dental resin composite materials should be established.

The aim of the study was to evaluate dentine surface after different chlorhexidine digluconate (CHG) application protocols.

## 2. Materials and Methods

Forty two caries-free human molars were used for the study. Prior to extraction, patients (aged 18–30 years) were informed about the use of the molars for research purposes and written consent was obtained. Third molars with fully developed roots extracted for orthodontic reasons were collected. Teeth were stored in 0.9% NaCl solution (exchanged daily) for a maximum of 1 week after extraction. The tooth surface was meticulously cleaned by removing soft tissues and debris with the use of a slow-speed (40,000 rpm) hand-piece and diamond disc until smooth enamel/cement surface was obtained. The study used dentinal samples prepared from vestibular surfaces of teeth. First, vestibular enamel was removed with a diamond shoulder bur (grit size 100 μm), then the tooth crown was cut along the mesio-distal axis, 2 mm beneath dentino-enamel junction, and the root was cut perpendicularly. After removal of pulpal residues with a slow-speed and round steel bur, dentine was wet polished with SiC abrasive papers: P180 followed by P400. The dentine surface was carefully and thoroughly rinsed with water and dried. Dentinal samples were randomly divided into 14 study groups. For each group, three samples were prepared (42 total). Study groups are presented in Table 1.

Dentine sample surfaces were sputter coated with gold and examined using scanning electron microscopy with an energy disperse spectrometer (SEM–EDS) (Hitachi S-3000N, Hitachi, Chiyoda, Tokyo, Japan) system, allowing changes in their morphology and chemical composition to be determined. The representatives SEM images for each study group were captured at 200×, 1000×, and 5000× magnification. In addition, the dispersive spectroscopic analysis of the dentine surface was performed.

## 3. Results

**Figure 1:**
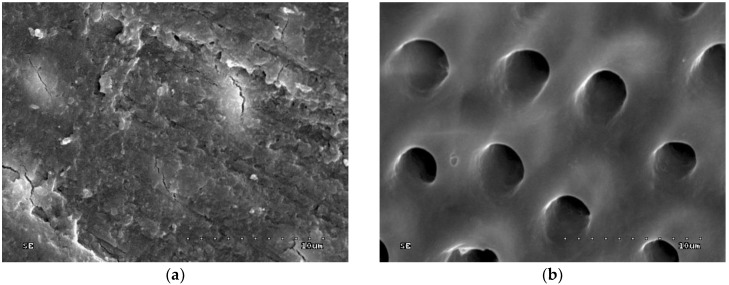
Scanning electron microscopy (SEM) image of dentine surface: (**a**) Prepared with bur, unetched–control group (group 1), 1000×; (**b**) etched with 36% orthophosphoric acid—group 2, 5000×.

Figure 1a shows dentine surface covered with a thick and homogenous smear layer. The dentinal tubule orifices are not visible. Scratches on dentine surface were created by mechanical preparation (rotary movements of a bur). The groove arrangement reflects the position of the rotary instrument towards the prepared surface. The surface is relatively coarse due to the presence of grooves, while the cracks are the result of the sample preparation for the SEM study.

Figure 1b shows dentine surface etched with 36% orthophosphoric acid for 15 s; dentinal tubule orifices are visible (superficial minerals from intertubular dentine removed) and smear layer plugs and debris are removed. Moreover, margins of dentinal tubule orifices are rounded, which indicates the superficial demineralization of peritubular dentine. Areas of dentine among dentinal tubule orifices (intratubular dentine) are smoothed, which may prove superficial demineralization of intertubular dentine.

**Figure 2:**
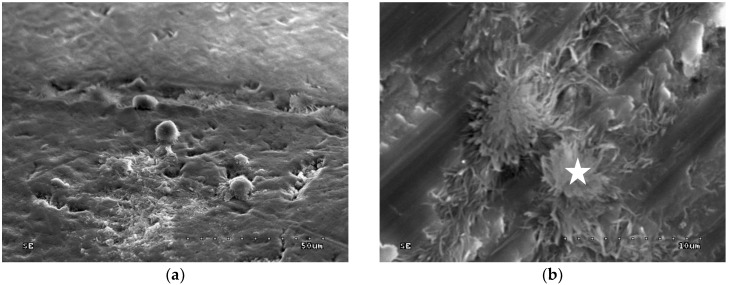
SEM image of dentine after application of 2.0% CHG for 30 s and drained—group 4: (**a**) 1000×; (**b**) 5000×. Star indicates the location, where the chemical analysis was performed.

In groups 3 and 4 (0.2% CHG or 2.0% CHG for 30 s on dentine surface and drained with sterile gauze), multiple and singular deposits loosely deployed on the dentine surface were observed (Figure 2a). Those deposits are found in pits created via mechanical preparation, as well as on the smooth surface of the sample (Figure 2a,b).

**Figure 3:**
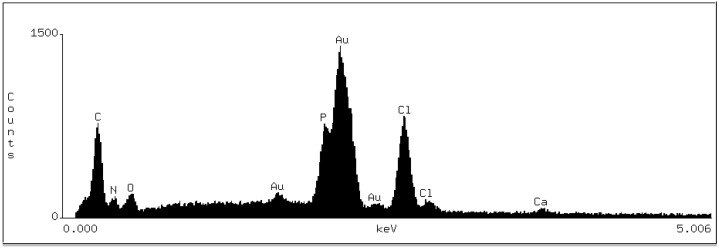
Dispersive spectroscopic analysis taken from the location indicated with the star in Figure 2b, where C—carbon, N—nitrogen, O—oxygen, Au—gold, P—phosphate, Cl—chloride, Ca—calcium.

Figure 3 shows the dispersive spectroscopic analysis obtained from the location indicated with a star on the Figure 2b.

The spectrogram showed the peaks of the following elements: Carbon (C), nitrogen (N), oxygen (O), gold (Au), phosphate (P), chloride (Cl), and calcium (Ca). The peaks of P and Ca indicate the presence of the compounds of these elements (e.g., calcium hydroxyapatites and/or phosphates), which are the components of dentine underlying the tested deposit. The presence of O peaks might be explained by the fact that this element is also a component of the hydroxyapatite. The Au peak showed in the analysis is due to the sputter deposition of gold on the sample surface. C, N, and Cl are the constituents of chlorhexidine digluconate with the formula [–(CH_2_)_3_NHC(=NH) NHC(=NH)NHC_6_H_4_Cl]_2_. The chemical analysis of the deposits may indicate the presence of CHG compound in the tested deposit.

**Figure 4:**
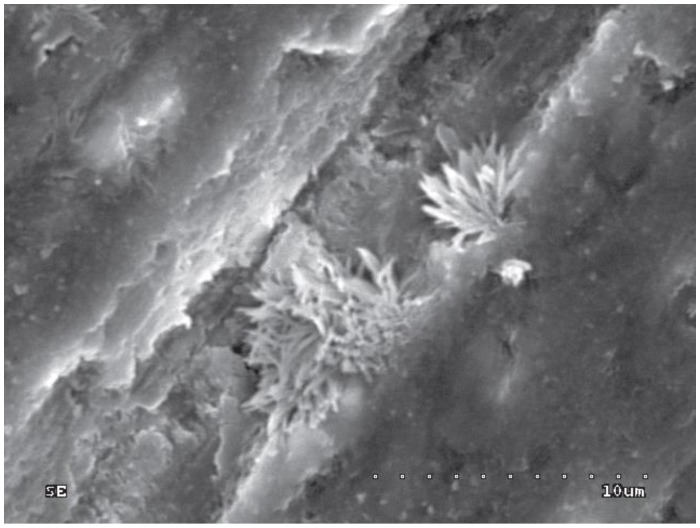
SEM image of dentine surface after application of 0.2% CHG for 30 s and rinsing with water–group 5, 5000×.

In groups 5 and 6 (0.2% CHG and 2.0% CHG applied for 30 s and rinsed with water), the smear layer covering the surface sample and few CHG deposits only in pits created through diamond bur surface preparation were detected (Figure 4). Deposits were more numerous when a higher concentration of CHG was applied.

**Figure 5:**
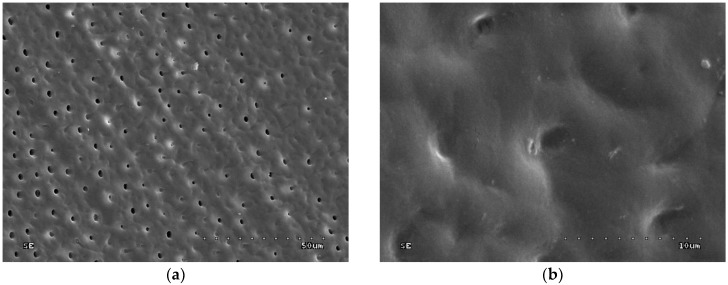
SEM image of dentine surface after application of 2.0% CHG for 30 s and acid etching—group 9: (**a**) 1000×; (**b**) 5000×.

**Figure 6:**
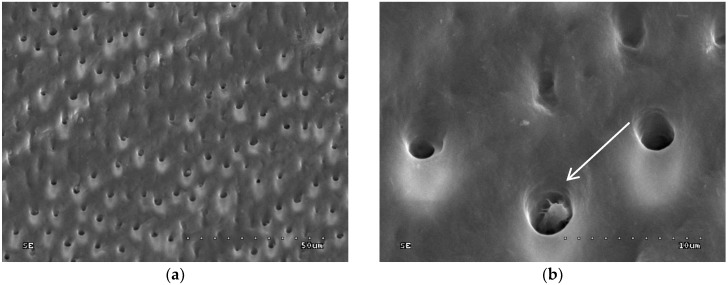
SEM image of dentine surface after application of 0.2% CHG for 30 s and acid etching—group 7: (**a**) 1000×; (**b**) 5000×. Arrow indicates peritubular dentine residues.

**Figure 7:**
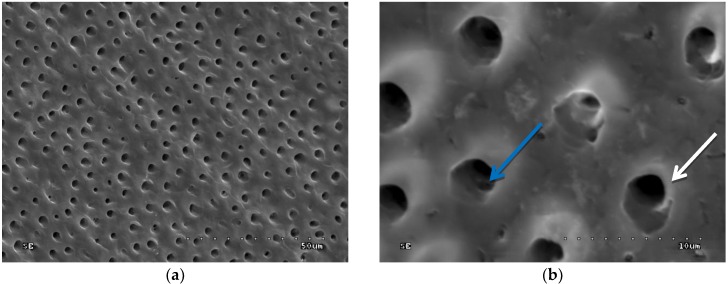
SEM image of dentine surface after application of 0.2% CHG for 15 s and acid etching—group 8: (**a**) 1000×; (**b**) 5000×. Blue arrow indicates open lateral dentinal tubule. White arrow—peritubular dentine residues.

**Figure 8:**
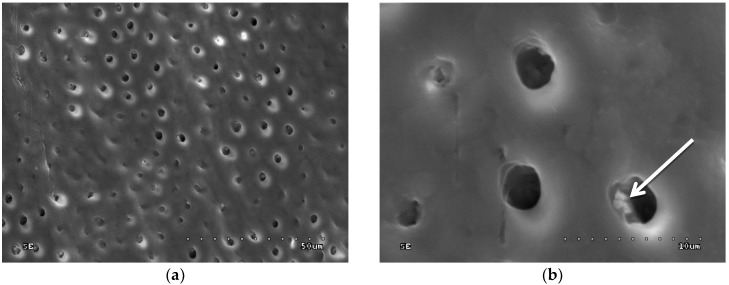
SEM image of dentine surface after application of 2.0% CHG for 15 s and acid etching—group 10: (**a**) 1000×; (**b**) 5000×. White arrow indicates peritubular dentine residue.

In groups 7 to 10 (CHG was applied, drained with sterile gauze, and acid-etched), the sample surface was inhomogeneous: Dentinal orifices were exposed to a variable extent and dentine surface was locally covered with smear layer and smear layer plugs. The degree of dentinal orifice exposure was the lowest for group 9 (2.0% CHG for 30 s), higher for groups 7 (0.2% CHG for 30 s) and 10 (2.0% CHG for 15 s), while the highest for group 8 (0.2% CHG for 15 s) (Figure 5, Figure 6, Figure 7 and Figure 8). In Figure 7, peritubular dentin was not completely removed, and most of the dentinal tubule orifices remained obliterated. Residues of peritubular dentine (white arrow) were visible in Figure 6, Figure 7 and Figure 8, while in Figure 7 the open orifice of the lateral dentinal tubule could also be observed (blue arrow).

**Figure 9:**
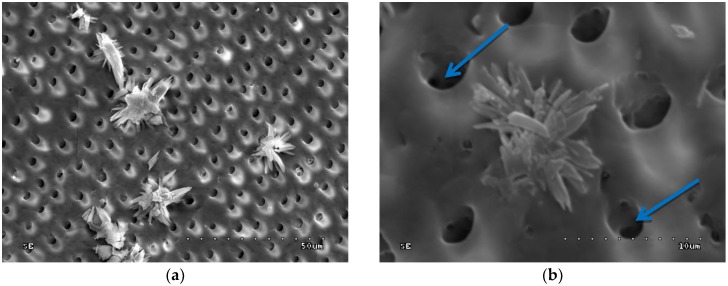
SEM image of acid-etched dentine surface after application of 2.0% CHG for 30 s—group 13: (**a**) 1000×; (**b**) 5000×. Blue arrows show open lateral dentinal tubules.

**Figure 10:**
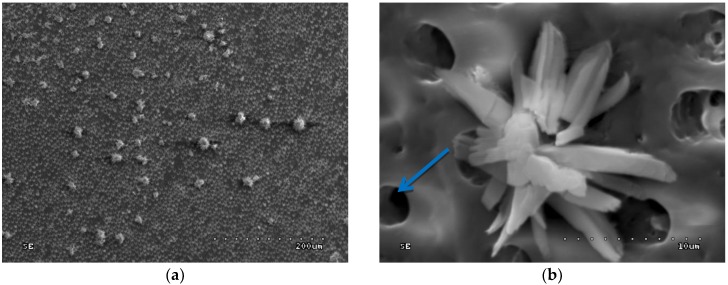
SEM image of acid-etched dentine surface after application of 2.0% CHG for 15 s—group 14: (**a**) 200×; (**b**) 5000×. Blue arrow shows open lateral dentinal tubule.

In groups 11–14 (acid etched, rinsed with water and drained, then CHG applied), orifices of dentinal tubules (removed intertubular dentine) and removed smear plugs were observed. The margins of dentinal orifices were rounded, which indicates the superficial demineralization of peritubular dentine. Moreover, CHG deposits were loosely dispersed on the surface of etched dentine (Figure 9a and Figure 10a) and inside the orifices of dentinal tubules (Figure 9b and Figure 10b). CHG deposits were the most numerous in group 13 (2.0% CHG for 30 s) (Figure 9), less numerous in groups 11 (0.2% CHG for 30 s) and 14 (2.0% CHG for 15 s) (Figure 10), while the lowest number was present in group 12 (0.2% CHG for 15 s). In Figure 9 and Figure 10, open orifices of lateral dentinal tubules could be observed (blue arrows).

## 4. Discussion

Unfortunately, there is limited literature on the comprehensive analysis of dentine surface treatment (water rinsing, etching, CHG application). Scanning electron microscopy, along with chemical microanalysis/X-ray spectroscopy, allowed us to investigate how different methods of CHG application can modify the dentine surface, and to establish the elemental composition of the deposits. In particular, the aim was to define whether CHG will remain on the dentine surface after water rinsing or drying, and whether it depends on application time and antiseptic concentration. Moreover, the influence of CHG application on dentine morphology and etching pattern is worth investigating. The modification of dentine surface prior to or during adhesive procedures may influence the strength and durability of the adhesive interface.

Available literature describes either the dentine covered with a smear layer or its removal after etching. After the mechanical preparation, the smear layer covering the regular dentine is characterized by a relatively smooth surface with a consistent layer of dentinal debris [38]. Underneath the smear layer lies the coarse dentine surface, covered with numerous grooves [39]. Uneven surface corresponds with the type and position of rotary instruments in relation to the prepared surface. After etching the dentine with phosphate acid, the smear layer is removed and the lumens of dentinal orifices are opened. The widening of dentinal orifice lumens can be observed because peritubular dentine is partially removed. Moreover, the superficial demineralization of intertubular dentine and removal of intratubular dentine with smear plugs are observed [40,41,42,43]. The abovementioned results confirm the present study.

In the present study (confirmed by EDS analysis), CHG was observed in the grooves only on the dentine surface rinsed with water. When CHG was dried, the CHG deposits covered the entire dentine sample surface. In addition, when higher concentrations of CHG were used on the unetched dentine surface, a higher concentration of CHG deposits was detected. Similar results were obtained by Di Hipólito et al. [44], who in an SEM/EDS study observed a higher concentration of Cl ions on the dentine surface when 2.0% CHX was applied, in comparison to 0.2% CHX application. Moreover, in the present study, slight removal of the smear layer was observed, regardless of the CHG concentration. Hiraishi et al. [45,46] also concluded that CHG does not successfully remove the smear layer. On the contrary, Castro et al. [34] observed partial removal of the smear layer with slight opening of dentinal tubule orifices. Such differences in the study results may be related to the intensity of rinsing the dentine surface with CHG.

In the present study, when CHG was applied on the dentine surface and drained prior etching, partial removal of the smear layer was observed and dentinal tubule orifices were opened. The degree of smear layer removal depended on the application time and concentration of disinfecting agent. The dentinal tubule orifices were most opened after 0.2% CHG application for 15 s, while they were narrowest after 30 s application of 2.0% CHG. When CHG was applied on etched dentine surface, the pattern on the surface was typical for orthophosphoric acid etched dentine, but enriched with loosely disposed CHG deposits on dentine surface and in the orifices. Furthermore, the same relationship between CHG deposit concentration and application period and disinfectant concentration were observed. Similar results were obtained by Perdigao et al. [47]. It may be hypothesized that CHG may form an additional retention in the hybrid layer for the bonding system with dentine, increasing the adhesive area and causing short-, as well as long-term, increases in the bond strength. The increase in strength may be strongly related to the degree of CHG deposit retention to the dentine.

The application of 2.0% CHG for 40 s followed by drying the dentine surface prior to Clearfil SE Bond usage did not influence marginal adaptation of the bonding area tested with methylene blue diffusion method [48]. Ercan et al. [30] observed fully developed resin stripes (Prime&Bond NT) penetrating into dentinal tubules after etching of dentine, modified with gel containing 1.0% CHX. Hebling et al. [12] and Carrilho et al. [13] observed hybrid layer with regular and homogenous collagen matrix, produced with 2.0% CHG and Adper Single Bond, while in control group progression of collagen degradation was observed. It can be assumed that the application of CHG prior to adhesive treatment may lead to an increase in adhesive bonding durability. Based on the abovementioned study results, the application of CHG on etched dentine surface is recommended.

Loguercio et al. [49] reported that 2.0% CHG applied on acid-etched dentine was still present in the adhesive interface after five-year follow-up.

## 5. Conclusions

The concentration of CHG and its application time influenced the amount of CHG deposits and the degree of smear layer removal from the dentine surface. Rinsing the unetched dentine with water after the application of CHG removed most of the CHG deposits from the surface. The application of 0.2% CHG for 15 s, followed by draining, on either etched or unetched dentine surface, effectively removed the smear layer, leaving the surface enriched with CHG deposits.

## Figures and Tables

**Table 1:** Dentine surface treatment (study groups).

Group Number	Dentine Surface Treatment			
1	No surface treatment			
2	15 s acid etching			
3	0.2% CHG for 30 s	drained with sterile gauze		
4	2.0% CHG for 30 s			
5	0.2% CHG for 30 s			rinsed with water for 10 s and dried
6	2.0% CHG for 30 s			
7	0.2% CHG for 30 s	drained with sterile gauze	15 s acid etching	rinsed with water for 10 s and dried
8	0.2% CHG for 15 s			
9	2.0% CHG for 30 s			
10	2.0% CHG for 15 s			
11	15 s acid etching	rinsed with water for 10 s and dried	0.2% CHG for 30 s	drained with sterile gauze
12	15 s acid etching		0.2% CHG for 15 s	
13	15 s acid etching		2.0% CHG for 30 s	
14	15 s acid etching		2.0% CHG for 15 s	
